# Composition Retention Metrics Reveal Route-Specific Controls of Glucose Release from Pretreated Kenaf Core

**DOI:** 10.1021/acsomega.6c00476

**Published:** 2026-04-14

**Authors:** Yitong Niu, Ying Ying Tye, Chee Keong Lee, Cheu Peng Leh

**Affiliations:** † Bioresource Technology Division, School of Industrial Technology, 26689 Universiti Sains Malaysia, USM, Gelugor 11800, Penang, Malaysia; ‡ Bioprocess Technology Division, School of Industrial Technology, Universiti Sains Malaysia, USM, Gelugor 11800, Penang, Malaysia

## Abstract

Lignocellulosic sugar production relies on pretreatments that improve enzymatic digestibility without excessive loss of recoverable solids and carbohydrates. This study benchmarks autohydrolysis, dilute-acid (H_2_SO_4_), and alkaline (NaOH) pretreatments of kenaf core and introduces a retention-aware interpretation framework based on two complementary composition descriptors for cellulose, hemicellulose, and lignin: a relative composition ratio (*R*) that captures enrichment/depletion in the recovered solid and an absolute retention index (AR) that incorporates solid recovery to quantify true feedstock-basis retention. Glucose release was evaluated using paired yield bases (recovered-solid and raw-feedstock) together with solid yield and cellulose conversion to decouple digestibility from composition-driven enrichment effects. Across routes, recovered-solid-basis glucose yield reached 38.8% (autohydrolysis) and 37.6% (dilute acid) at moderate solid yields, whereas alkaline pretreatment combined higher recovery (up to 72.4%) with moderate-to-high digestibility (13.6–37.6%). Raw-feedstock-basis glucose yield ranged from 1.20–23.30% (autohydrolysis), 1.30–19.70% (dilute acid), and 8.70–18.00% (alkaline), showing that mass loss can offset apparent gains in digestibility. Route-resolved LOESS trends and quadratic response surfaces identify hemicellulose depletion as the most consistent predictor of glucose release, while lignin enrichment is not transferable across chemistries; AR-based lignin retention becomes a graded separator primarily under alkaline conditions. The results support routine paired reporting of R/AR metrics with solid yield, yield basis, and cellulose conversion for defensible cross-route comparison.

## Graphical abstract

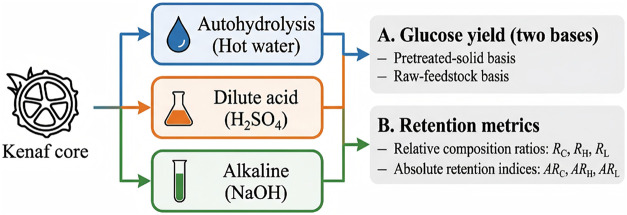

## 1 Introduction

Lignocellulosic biomass is increasingly viewed as a strategic feedstock for producing renewable fuels and biobased chemicals because it can supply fermentable sugars without competing directly with food resources.^1–4^ Realizing this potential, however, depends on efficiently converting the structural carbohydrates locked in plant cell walls into monomeric sugars that can be upgraded through biochemical routes.^5,6^ In native biomass, cellulose is protected by a complex, hierarchical matrix in which hemicellulose and lignin constrain water uptake, limit enzyme accessibility, and promote nonproductive adsorption, collectively imposing strong recalcitrance.^7–9^ As a result, pretreatment remains a critical enabling step for lignocellulosic sugar platforms, because it governs both the extent to which polysaccharides become accessible for hydrolysis and the extent to which solids and carbohydrates are retained for downstream conversion.^10,11^

Kenaf is an established fiber crop with a well-developed value chain, and its core fraction represents an abundant lignocellulosic resource that is often underutilized relative to the bast fiber.^12^ From a bioconversion perspective, kenaf core offers an attractive substrate for sugar-based processing, but it exhibits the same structural barriers typical of lignified agricultural residues, so pretreatment is required to unlock enzymatic hydrolysis.^13,14^ Importantly, the relevance of kenaf core as a feedstock is not determined by digestibility alone. Pretreatment also dictates solid recovery and component retention, which together control how much fermentable carbohydrate remains available on an original-feedstock basis and, by extension, the practical yield that can be achieved in an integrated process.^15,16^ A comparative evaluation that considers both hydrolysis performance and recovery-linked compositional change is therefore necessary to position kenaf core within lignocellulosic sugar platforms.^17,18^

Among the pretreatment strategies commonly applied to lignocellulosic feedstocks, autohydrolysis, dilute-acid pretreatment, and alkaline pretreatment represent three chemically distinct routes that modify the cell-wall matrix through different dominant reactions.^19,20^ Autohydrolysis relies on pressurized hot water and the in situ generation of hydronium ions, which promotes hemicellulose solubilization and partial depolymerization, thereby increasing porosity and facilitating subsequent hydrolysis.^21–23^ Dilute-acid pretreatment intensifies these carbohydrate-directed reactions, accelerating hemicellulose hydrolysis and deacetylation but also increasing the likelihood of sugar degradation and secondary reactions that can alter lignin structure and its distribution on fiber surfaces.^24–26^ In contrast, alkaline pretreatment targets lignin–carbohydrate linkages and lignin solubilization, often reducing lignin shielding and nonproductive enzyme binding while leaving a larger fraction of hemicellulose in the solid.^27–29^ Because these routes shift hemicellulose and lignin in fundamentally different ways, the same change in measured solid composition can reflect different structural states and, consequently, different hydrolysis responses across pretreatment chemistries.

Despite decades of pretreatment research, cross-route comparisons remain difficult because the same performance and composition descriptors can carry different meanings across hydrothermal/acid and alkaline chemistries.^11,30^ Recovered-solid-basis yields quantify digestibility of the remaining substrate, whereas feedstock-basis yields quantify conversion after pretreatment losses; when these bases are reported or interpreted in isolation, it becomes unclear whether changes in performance are driven by accessibility gains, carbohydrate removal, or both.^31,32^ Composition-based explanations face a related limitation. Increases in the cellulose or lignin fractions of recovered solids may reflect true retention, preferential solubilization of other components, or route-specific artifacts, and therefore cannot be treated as universally comparable “enrichment” signals across pretreatments.^24,25^ Moreover, single-variable comparisons can obscure nonlinearity and interaction effects that govern hydrolysis outcomes under different chemistries.^16^

Kenaf core was used as a single, well-defined lignocellulosic substrate to enable a chemistry-to-chemistry comparison of autohydrolysis, dilute-acid, and alkaline pretreatments within controlled operating windows. The study adopts paired yield reporting, in which glucose release is expressed on both a recovered-solid basis and an original-feedstock basis together with solid yield, so that digestibility and pretreatment losses can be interpreted in a coupled manner. As summarized in Scheme 1, compositional change is analyzed using two complementary retention descriptors for cellulose, hemicellulose, and lignin. A relative composition ratio captures enrichment or depletion within the recovered solid, whereas a feedstock-basis retention index incorporates solid recovery to distinguish true component retention from concentration effects driven by mass loss. To avoid implying a universal composition–hydrolysis function across mechanistically distinct chemistries, trends are evaluated using route-aware, nonparametric smoothing and interaction mapping through quadratic response surface modeling. This retention-aware, route-comparable workflow is intended to strengthen the interpretability of pretreatment data sets and to support more defensible pretreatment selection for lignocellulosic sugar production.

**Scheme 1:**
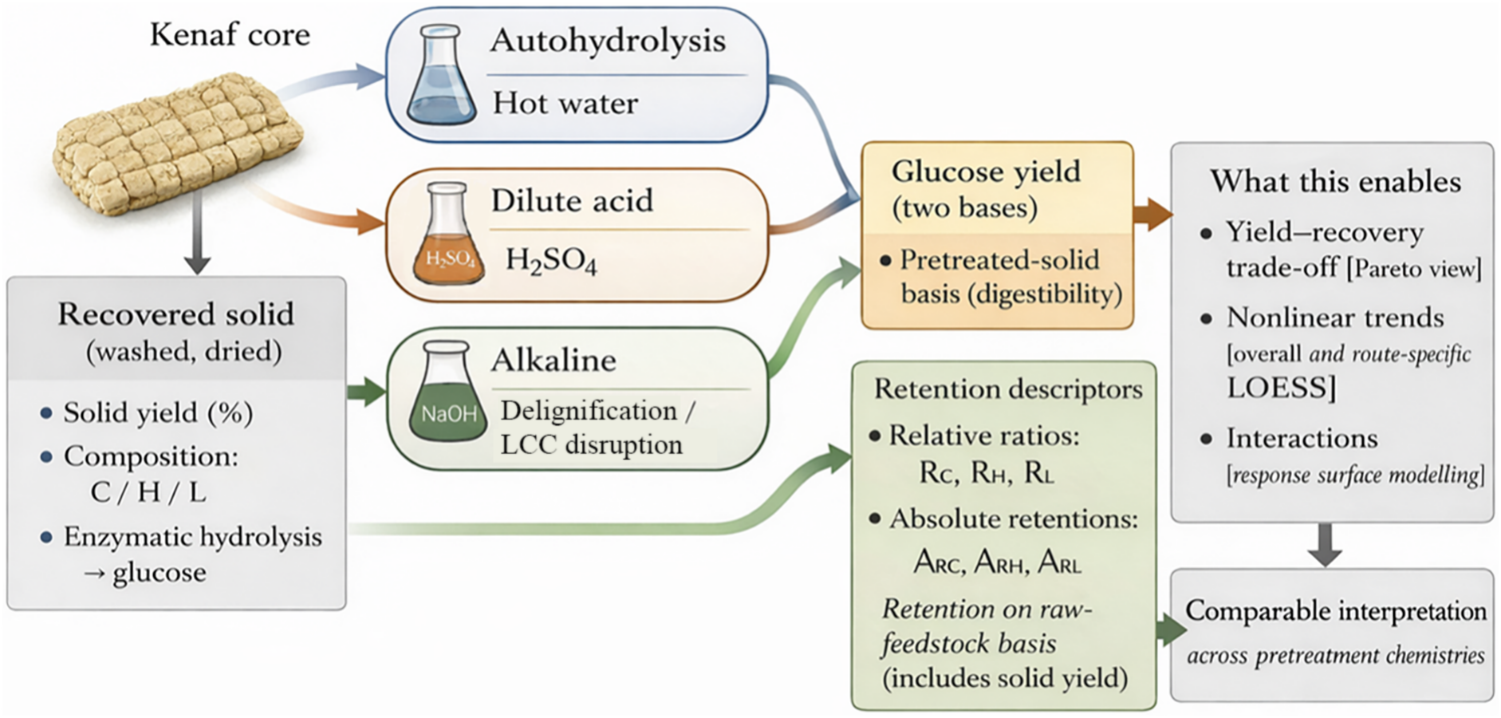
Retention-Aware Comparison of Pretreatment Routes for Kenaf Core (Created by the Authors)

## 2 Materials and Methods

### 2.1 Feedstock

Kenaf core (nominal particle length 3–6 mm) was obtained as a single bulk lot from the National Kenaf and Tobacco Board (Malaysia) and was homogenized to prepare a representative composite feedstock used throughout this study. The material was thoroughly washed with tap water to remove adhering dust and soluble impurities and then air-dried to constant mass. The dried kenaf core was gently homogenized to obtain a representative composite batch, and visible nonfibrous contaminants were manually removed. The prepared feedstock was stored in sealed polyethylene bags at room temperature in the dark until further use.^33^ Unless otherwise stated, all masses and compositions reported in this study are expressed on an oven-dry basis.

The moisture content of the air-dried kenaf core was determined prior to each pretreatment run using a benchtop moisture analyzer (MA35, Sartorius, Germany) operated at 105 °C until a constant mass was recorded.^34^ Approximately 1.0 g of representative material was sampled from the prepared feedstock bag for each measurement, and determinations were performed in triplicate. Moisture content was calculated as

1 $$MC\;\left(\%\right)=\frac{m_{\mathrm{AD}}-m_{\mathrm{OD}}}{m_{\mathrm{AD}}}\times 100$$

where *m*_AD_ is the mass of the sample in the air-dry state and *m*_OD_ is the mass after drying to constant weight (oven-dry state). Air-dry mass was converted to oven-dry mass as

2 $$m_{\mathrm{OD}}=m_{\mathrm{AD}}\times \left(1-\frac{MC}{100}\right)$$

The measured moisture content was used to correct the charged biomass mass so that an identical oven-dry (OD) loading was applied in all pretreatment experiments.

### 2.2 Pretreatment Procedures

Autohydrolysis, dilute-acid (H_2_SO_4_), and alkaline (NaOH) pretreatments were performed in a 4 L stationary stainless-steel digester (NAC Autoclave Co., Japan) equipped with a microcomputer-controlled thermocouple.^35^ A fixed solid-to-liquor ratio of 1:12 (w/w) was used for all runs.^36^ For autohydrolysis, slurries were heated to the target temperature (150, 170, or 180 °C) and held for the prescribed residence time (30, 45, or 60 min) before being rapidly quenched in a water bath.

After pretreatment, the solid fraction was recovered by filtration, washed thoroughly with deionized water until the wash filtrate approached neutral pH, and oven-dried to constant mass for solid-yield determination on an oven-dry basis. The corresponding filtrates were collected and cooled to room temperature for pH measurement. The pretreatment conditions explored for each pretreatment route are summarized in Table 1.

**Table 1:** Conditions for the Different Pretreatment Methods

pretreatment methods	temperature (°C)	residence time (min)	reagent concentration (%, w/w)
Autohydrolysis	150–180	30–60	N/A
Dilute acid (H_2_SO_4_)	120–140	45–90	1.0–2.0
Alkaline (NaOH)	100–140	45–60	1.0–3.0

### 2.3 Chemical Composition Analysis

Untreated and pretreated kenaf samples were gently milled under air-dry conditions and passed through a 2.0 mm sieve prior to analysis. For the untreated material, extractives were removed following TAPPI T 204 using an ethanol–toluene solvent system. Pretreated solids were treated as extractives-free because of the prior contact with pretreatment liquor and subsequent extensive washing.

Acid-insoluble (Klason) lignin was determined according to TAPPI T 222, and acid-soluble lignin was quantified spectrophotometrically following TAPPI UM 250.^37^ Holocellulose was obtained using the sodium chlorite (Wise) method, and cellulose fractions were determined by the graded-alkali procedure (JIS 8101).^38^ In this study, cellulose was defined as α-cellulose, hemicellulose as the combined β- and γ-cellulose fractions, and total lignin as the sum of Klason lignin and acid-soluble lignin. All compositional results are reported on an oven-dry basis.

### 2.4 Enzymatic Hydrolysis

Enzymatic hydrolysis was performed using a commercial cellulase preparation (Celluclast 1.5 L, Novozymes A/S, Denmark).^39^ Reactions were set up in 250 mL Erlenmeyer flasks containing pretreated kenaf core at 2.5% (w/v) solids in 0.05 M citrate buffer (pH 4.8). The enzyme dosage was fixed at 70 FPU g^–1^ substrate. Flasks were tightly capped and incubated in a shaking water bath at 50 °C and 100 rpm for 48 h.^40^

Hydrolysis was terminated by boiling aliquots for 10 min, followed by centrifugation at 4000 rpm for 10 min to remove unhydrolysed residues. The supernatants were collected and stored at −20 °C until glucose analysis. All hydrolysis experiments were conducted at least in duplicate, with buffer blanks and enzyme controls included to correct for background.

### 2.5 Glucose Quantification

Glucose concentrations in the enzymatic hydrolysates were quantified by high-performance liquid chromatography (HPLC; Agilent Technologies) equipped with a Hi-Plex Ca column (300 × 7.7 mm). Distilled–deionized water was used as the mobile phase at a flow rate of 0.6 mL min^–1^. Prior to analysis, hydrolysate samples were filtered through 0.22 μm syringe filters, and 20 μL aliquots were injected. Glucose was quantified by external calibration using standard glucose solutions. All measurements were performed at least in duplicate, and the resulting concentrations were used for subsequent yield calculations.

### 2.6 Calculations and Definition of Composition Retention Metrics

Solid yield was defined on an oven-dry basis as the fraction of dry biomass recovered after pretreatment relative to the initial dry mass charged to the reactor

3 $$\mathrm{solid}\;\mathrm{yield}\;\left(\%\right)=\frac{m_{\mathrm{pretreated}}}{m_{\mathrm{raw}}}\times 100$$

where *m*_raw_ is the initial oven-dry mass of kenaf core and *m*_pretreated_ is the oven-dry mass of the recovered solid after washing and drying to constant mass.

Glucose yield was calculated in two forms to support distinct analytical purposes. The pretreated-basis glucose yield was defined as

4 $${\mathrm{glucose}\;\mathrm{yield}}_{\mathrm{pretreated}}\;\left(\%\right)=\frac{m_{\mathrm{glucose}}}{m_{\mathrm{pretreated}}}\times 100$$

where *m*_glucose_ is the mass of glucose released after enzymatic hydrolysis. For comparison to the original feedstock, the untreated-basis glucose yield was computed as

5 $${\mathrm{glucose}\;\mathrm{yield}}_{\mathrm{untreated}}\;\left(\%\right)={\mathrm{glucose}\;\mathrm{yield}}_{\mathrm{pretreated}}\;\left(\%\right)\times \frac{\mathrm{solid}\;\mathrm{yield}\;\left(\%\right)}{100}$$

To decouple enzymatic digestibility from compositional enrichment of the recovered solid, cellulose conversion was also calculated as the fraction of glucan in the pretreated solid that was released as glucose:

6 $$glu\cos e\;conversion\;\left(\%\right)=\frac{m_{\mathrm{glucose}}}{1.111\times m_{\mathrm{pretreated}}\times f_{\mathrm{cellulose}}}\times 100$$

where *f*_cellulose_ is the cellulose mass fraction in the pretreated solid (oven-dry basis), and 1.111 is the stoichiometric factor converting anhydroglucose units in cellulose to glucose.

To link chemical composition with hydrolysis performance, two sets of composition retention metrics were defined for cellulose (C), hemicellulose (H), and lignin (L) based on the measured mass fractions of each component in the raw biomass (*X*_raw_) and in the pretreated solid (*X*_pretreated_). The relative composition ratio (*R*) was defined as

7 $$R_{C/H/L}\;\left(\%\right)=\frac{X_{C/H/L,\mathrm{pretreated}}}{X_{C/H/L,\mathrm{raw}}}\times 100$$

The absolute composition retention index (AR) incorporated solid recovery to account for mass losses during pretreatment and was defined as

8 $$AR_{C/H/L}\left(\%\right)=\frac{X_{C/H/L,\mathrm{pretreated}}\times \mathrm{solid}\;\mathrm{yield}\;\left(\%\right)}{X_{C/H/L,\mathrm{raw}}}$$

In subsequent analyses, *R*_C_, *R*_H_, and *R*_L_ were used to represent cellulose enrichment, hemicellulose depletion, and lignin enrichment in the recovered solid, whereas AR_C_, AR_H_, and AR_L_ were used to represent the corresponding absolute retentions on an original-feedstock basis.

## 3 Results and Discussion

As reported in Tables 2, 3 and 4, the three pretreatment routes occupy distinct composition–recovery spaces, with the strongest separation arising from hemicellulose depletion and lignin behavior. In addition to reporting glucose yield on both a recovered-solid basis and a raw-feedstock basis, Tables 2–4 also report cellulose conversion to decouple enzymatic digestibility from composition-driven enrichment effects in the recovered solids. Autohydrolysis recovered 59.8–77.1% of solids and produced materials with RC of 101.42–130.07, RH of 2.14–57.11, and consistently high lignin enrichment (RL of 161.08–227.09); the corresponding glucose yield ranged from 1.60% to 38.80% on a recovered-solid basis and from 1.20% to 23.30% on a raw-feedstock basis. Dilute-acid pretreatment showed a comparable solid-yield range (48.2–66.3%) but drove hemicellulose depletion more strongly (RH of 0–19.92) alongside the highest lignin enrichment (RL of 200.49–256.16); glucose yield ranged from 2.00% to 37.60% on a recovered-solid basis and from 1.30% to 19.70% on a raw-feedstock basis. In contrast, alkaline pretreatment recovered 48.0–72.4% of solids and shifted lignin in the opposite direction, with substantially lower RL values (86.21–161.08) and moderate RH values (16.22–36.39); its glucose yield distribution was less skewed toward very low values, ranging from 13.60% to 37.60% on a recovered-solid basis and from 8.70% to 18.00% on a raw-feedstock basis.

**Table 2:** Chemical Composition and Enzymatic Hydrolysis of Autohydrolysis Kenaf Fibre at Different Pretreatment Conditions

operating conditions									
Time (min)	30	30	30	45	45	45	60	60	60
Temperature (°C)	150	170	180	150	170	180	150	170	180
Solid Yield (%)^a^	77.1	62.3	70.8	65.7	60	65.5	66.6	59.8	68.2
Pretreated (%)									
Chemical composition									
Cellulose	43.80	54.39	49.90	53.50	56.18	53.88	54.28	54.11	53.15
Hemicellulose	28.60	5.25	11.40	10.50	1.38	8.69	10.48	1.79	8.15
Lignin	32.70	42.90	40.90	40.90	45.30	43.80	38.20	42.90	46.10
Composition ratio									
R_C_ ^b^	101.42	125.92	115.53	123.88	130.07	124.74	125.68	125.29	123.05
*R*_H_ ^b^	57.11	8.48	20.91	17.86	2.14	14.74	18.08	2.77	14.40
R_L_ ^b^	161.08	211.33	201.48	201.48	223.15	215.76	188.18	211.33	227.09
Glucose yield	1.60	25.20	4.40	5.20	38.80	8.80	6.80	32.40	10.80
Cellulose conversion	3.29	41.7	7.94	8.75	62.16	14.7	11.28	53.9	18.29
Untreated (%)									
Chemical composition									
Cellulose	33.77	33.88	35.33	35.15	33.71	35.29	36.15	32.36	36.25
Hemicellulose	22.05	3.27	8.07	6.90	0.83	5.69	6.98	1.07	5.56
Lignin	25.20	26.70	28.90	26.80	27.20	28.70	25.40	25.70	31.40
Composition ratio									
AR_C_ ^c^	78.19	78.45	81.80	81.39	78.04	81.70	83.71	74.92	83.92
A*R*_H_ ^c^	74.07	13.61	29.53	27.18	3.57	22.50	27.15	4.63	21.12
AR_L_ ^c^	>99	>99	>99	>99	>99	>99	>99	>99	>99
Glucose yield	1.20	15.70	3.10	3.40	23.30	5.80	4.50	19.40	7.40

a Solid yield (%) = mass of pretreated solid/mass of original feedstock.

b *R*_C/H/L_ (%) = pretreatment fraction of the component/initial fraction of the component.

c AR_C/H/L_ (%) = Component fraction in treated/raw × Solid yield.

**Table 3:** Chemical Composition and Enzymatic Hydrolysis of Acid Pretreatment Kenaf Fibre at Different Pretreatment Conditions

operating conditions												
Time (min)	45	45	45	60	60	60	90	90	90	90	90	90
Temperature (°C)	120	140	140	120	120	140	120	120	120	140	140	140
H_2_SO_4_ concentration (%)	1	1	2	1.5	2.0	1.5	1	1.5	2	1	1.5	2
Solid Yield (%)^a^	66.3	48.2	50.9	62.6	57.2	50	60.5	54.1	52.4	54.00	54.90	55.30
Pretreated (%)												
Chemical composition												
Cellulose	51.80	52.70	49.56	55.73	57.41	50.44	52.94	55.12	54.10	55.19	53.81	49.74
Hemicellulose	11.60	0.00	6.44	3.87	0.99	3.56	5.36	2.48	0.00	3.21	2.59	1.96
Lignin	40.70	50.80	52.00	45.90	49.60	50.70	46.40	47.00	52.00	51.00	49.50	51.00
Composition ratio												
R_C_ ^b^	119.93	122.02	114.75	129.02	132.92	116.78	122.57	127.63	125.26	127.78	124.58	115.15
*R*_H_ ^b^	19.92	0.00	8.49	6.28	1.47	4.62	8.40	3.47	0.00	4.49	3.69	2.81
R_L_ ^b^	200.49	250.25	256.16	226.11	244.33	249.75	228.57	231.53	256.16	251.23	243.84	251.23
Glucose yield	2.00	28.40	24.80	11.20	19.20	26.00	10.00	28.40	37.60	14.80	24.00	18.40
Cellulose conversion	3.48	48.51	45.04	18.09	30.10	46.40	17.00	46.38	62.56	24.14	40.15	33.30
Untreated (%)												
Chemical composition												
Cellulose	34.34	25.40	25.23	34.88	32.84	25.22	32.03	29.82	28.35	29.80	29.54	27.50
Hemicellulose	7.69	0.00	3.28	2.43	0.57	1.78	3.24	1.34	0.00	1.73	1.42	1.09
Lignin	27.00	24.50	26.50	28.70	28.30	25.20	28.10	25.50	27.20	27.50	27.20	28.20
Composition ratio												
AR_C_ ^c^	79.51	58.81	58.41	80.77	76.03	58.39	74.15	69.05	65.64	69.00	68.39	63.68
A*R*_H_ ^c^	30.05	0.00	16.68	10.03	2.57	9.23	13.89	6.41	0.00	8.32	6.72	5.09
AR_L_ ^c^	>99	>99	>99	>99	>99	>99	>99	>99	>99	>99	>99	>99
Glucose yield	1.30	13.70	12.60	7.00	10.90	13.00	6.10	15.40	19.70	8.00	13.20	10.20

a Solid yield (%) = mass of pretreated solid/mass of original feedstock.

b *R*_C/H/L_ (%) = pretreatment fraction of the component/initial fraction of the component.

c AR_C/H/L_ (%) = Component fraction in treated/raw × Solid yield.

**Table 4:** Chemical Composition and Enzymatic Hydrolysis of Alkaline Pretreatment Kenaf Fibre at Different Pretreatment Conditions

operating conditions										
Time (min)	45	45	45	45	45	60	60	60	60	60
Temperature (°C)	100	120	120	140	140	100	100	120	140	140
NaOH concentration (%)	2	1	3	1	3	1	3	2	2	3
Solid Yield(%)^a^	64.2	62.9	53.9	60.7	52.7	72.4	62.9	56.5	56.7	48
Pretreated (%)										
Chemical composition										
Cellulose	55.28	51.96	56.27	52.66	60.72	54.10	56.02	57.69	56.95	60.25
Hemicellulose	19.60	21.84	16.26	22.04	15.18	19.40	18.28	15.61	22.15	13.05
Lignin	32.20	24.00	27.80	29.40	26.90	29.00	32.70	29.10	24.90	17.50
Composition ratio										
R_C_ ^b^	127.98	120.29	130.27	121.93	140.59	125.25	129.71	133.56	131.86	139.50
*R*_H_ ^b^	32.59	35.59	22.70	34.64	20.72	36.39	29.78	22.85	32.53	16.22
R_L_ ^b^	158.62	118.23	136.95	144.83	132.51	142.86	161.08	143.35	122.66	86.21
Glucose yield	13.60	18.00	27.20	21.60	31.20	19.20	16.80	22.00	22.40	37.60
Cellulose conversion	22.13	31.18	43.48	63.89	46.22	31.94	27.01	34..33	35.40	56.16
Untreated (%)										
Chemical composition										
Cellulose	35.49	32.68	30.33	31.97	32.00	39.17	35.24	32.59	32.29	28.92
Hemicellulose	12.58	13.74	8.77	13.38	8.00	14.05	11.50	8.82	12.56	6.26
Lignin	20.70	15.10	14.90	17.90	13.10	21.00	20.60	16.50	14.10	8.40
Composition ratio										
AR_C_ ^c^	82.17	75.66	70.22	74.01	74.09	90.68	81.59	75.46	74.77	66.96
A*R*_H_ ^c^	50.76	56.58	42.12	57.08	39.32	50.26	47.34	40.44	57.36	33.79
AR_L_ ^c^	101.97	74.38	73.40	88.18	64.53	103.45	101.48	81.28	69.46	41.38
Glucose yield	8.70	11.30	14.70	13.10	16.40	13.90	10.60	12.40	12.70	18.00

a Solid yield (%) = mass of pretreated solid/mass of original feedstock.

b *R*_C/H/L_ (%) = pretreatment fraction of the component/initial fraction of the component.

c AR_C/H/L_ (%) = Component fraction in treated/raw × Solid yield.

### 3.1 Sugar Yield and Solid Yield Trade-Offs

Figure 1(a) reveals a clear multiobjective trade-off between enzymatic digestibility and solid recovery after pretreatment. Across all routes, the highest pretreated-solid-basis yields cluster at only moderate solid yields, while the highest solid yields can coincide with very low glucose release, indicating that maximizing recovery alone does not guarantee improved saccharification. Highlighting the nondominated solutions, the Pareto-optimal set traces two practically distinct pathways: one route emphasizes high digestibility at moderate recovery, exemplified by autohydrolysis reaching 38.8% glucose yield at 60.0% solid yield, whereas the other emphasizes higher solid recovery with moderate digestibility, exemplified by alkaline pretreatment retaining 72.4% solids while achieving 19.2% glucose yield.

**Figure 1:**
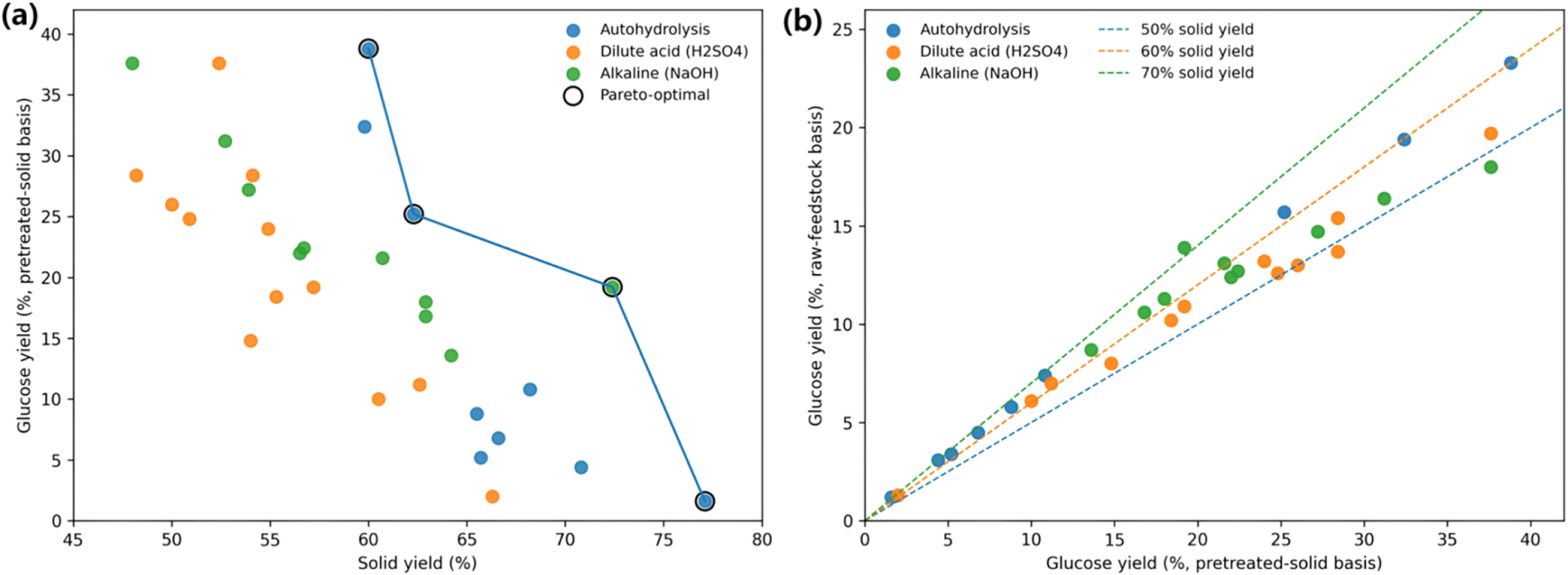
Trade-offs between glucose yield and solid recovery across pretreatment routes. (a) Glucose yield (Pretreated basis) versus solid yield. (b) Glucose yield (Pretreated basis) versus Glucose yield (Untreated basis).

At the high-digestibility end, autohydrolysis reaches the maximum pretreated-solid-basis glucose yield of 38.8% at a solid yield of 60.0%, while a second autohydrolysis point (25.2% at 62.3%) represents a less severe but still competitive compromise. At the high-recovery end, the highest solid yield (77.1%) coincides with only 1.6% glucose yield, showing that preserving solids without sufficient structural disruption can leave cellulose largely inaccessible. Between these extremes, alkaline pretreatment provides a high-recovery compromise at 72.4% solid yield with 19.2% glucose yield, indicating a distinct route to balanced performance.

Figure 1(b) clarifies how reporting basis changes the apparent ranking of pretreatment performance by making the conversion from pretreated-solid-basis yield to raw-feedstock-basis yield explicit. Because raw-basis yield is the product of pretreated-solid-basis yield and solid recovery, points with similar pretreated-solid-basis yields separate along the iso–solid-yield lines, so identical digestibility can translate into different feedstock-level sugar recovery when solid yield differs. For example, the same pretreated-solid-basis yield of 37.6% corresponds to a raw-basis yield of 19.7% under dilute acid at 52.4% solid yield, whereas under alkaline pretreatment it corresponds to 18.0% at 48.0% solid yield, illustrating the direct penalty imposed by lower recovery. Conversely, a moderate pretreated-solid-basis yield can produce a competitive raw-basis outcome when recovery is high, as shown by alkaline pretreatment achieving 13.9% raw-basis yield at 19.2% pretreated-solid-basis yield with 72.4% solid yield. These relationships show why a single yield basis is insufficient: pretreated-solid-basis yield reflects digestibility of the recovered material, while raw-basis yield captures feedstock-level conversion that is constrained by mass loss, and both are required for interpretable cross-route comparison.

### 3.2 Composition–Hydrolysis Relationships Revealed by LOESS Trends

Figure 2 summarizes the composition–hydrolysis relationships using route-specific LOESS fits, which avoids imposing a single pooled trend across mechanistically distinct pretreatment chemistries. Across routes, hemicellulose depletion remains the most consistent first-order correlate of high glucose release. For autohydrolysis and dilute-acid pretreatment, the highest pretreated-solid-basis glucose yields occur at the lowest hemicellulose-retention levels, with autohydrolysis reaching 38.8% at an *R*_H_ value of 2.14 and dilute acid reaching up to 37.6% at an *R*_H_ value of 0. By contrast, cellulose enrichment shows weaker discriminating power within each route. Although *R*_C_ stays elevated over the tested conditions, similar *R*_C_ values correspond to substantially different glucose yields, indicating that enrichment of cellulose fraction alone does not uniquely describe digestibility improvements.

**Figure 2:**
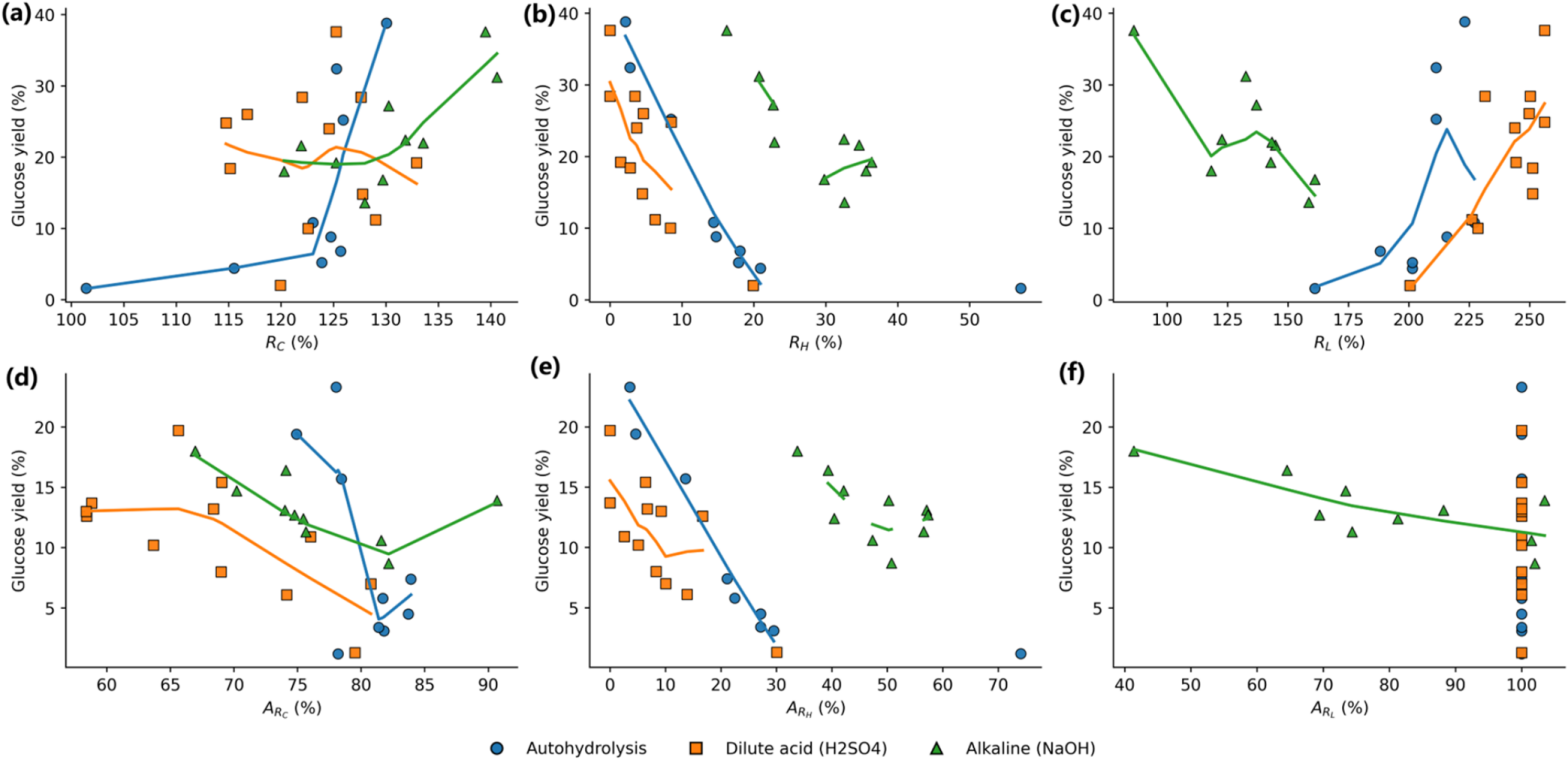
Composition retention metrics versus glucose yield across pretreatment routes: (a–c) *R*_C_, *R*_H_, and *R*_L_ versus glucose yield; (d–f) AR_C_, AR_H_, and AR_L_ versus glucose yield.

The route-specific fits further highlight that the same retention metric can map to different process meanings depending on pretreatment chemistry. For hemicellulose retention, autohydrolysis and dilute acid exhibit a steep increase in glucose yield as *R*_H_ decreases into the low-retention regime, after which additional hemicellulose removal produces smaller incremental gains. Alkaline pretreatment behaves differently: comparatively high glucose yields are attained without driving *R*_H_ to the extreme low end, and the highest yield of 37.6% occurs at an *R*_H_ value of 16.22, with most alkaline conditions remaining within an *R*_H_ range of approximately 20.7–36.4. This contrast is consistent with alkaline pretreatment achieving accessibility improvements through lignin modification/removal and fiber swelling, thereby reducing the requirement for near-complete hemicellulose depletion to reach high enzymatic response.

Lignin-related metrics show the strongest route dependence and therefore provide limited interpretability when treated as a single universal axis. Autohydrolysis and dilute-acid data sets remain confined to high lignin-enrichment ranges (*R*_L_ of 161.08–227.09 and 200.49–256.16, respectively) while still containing top-yielding conditions, which indicates that high apparent lignin fractions in the recovered solids do not, by themselves, preclude efficient hydrolysis in these two routes. In alkaline pretreatment, however, *R*_L_ spans a lower range (86.21–161.08), and high yields are concentrated toward the low-RL end, with the maximum yield (37.6%) occurring at an *R*_L_ value of 86.21, consistent with lignin removal acting as a stronger differentiator within the alkaline route.

Expressing composition change on a raw-feedstock basis using AR_C_, AR_H_, and AR_L_ adds interpretive value because these indices incorporate solid recovery and separate “enrichment by mass loss” from net component retention. This distinction is most relevant for dilute acid and autohydrolysis, where solid yield varies substantially (48.2–66.3% and 59.8–77.1%, respectively), so conditions with comparable *R*_C_ can retain different absolute amounts of cellulose on the feedstock basis. Accordingly, variation in raw-basis glucose yield (1.30–19.70% for dilute acid; 1.20–23.30% for autohydrolysis) is more defensibly interpreted against AR_C_ and AR_H_ than against *R*_C_ alone. For lignin, AR_L_ becomes practically informative primarily for alkaline pretreatment because it spans a genuinely low range (41.38–103.45%; plotted with a cap at 100% for clarity), along which raw-basis glucose yield varies from 8.70% to 18.00%. In contrast, autohydrolysis and dilute-acid conditions frequently show AR_L_ values above 100%, limiting within-route resolution and reinforcing that hemicellulose-depletion metrics dominate separation of hydrolysis outcomes in those two routes.

### 3.3 Interaction Patterns Captured by Response Surface Modeling

Quadratic response surface modeling was used to formalize the nonlinear and route-dependent patterns suggested by the LOESS trends and to identify interaction structures that cannot be resolved from single-metric relationships. Figure 3 visualizes the fitted response surfaces for glucose yield across pairwise combinations of the composition metrics.

**Figure 3:**
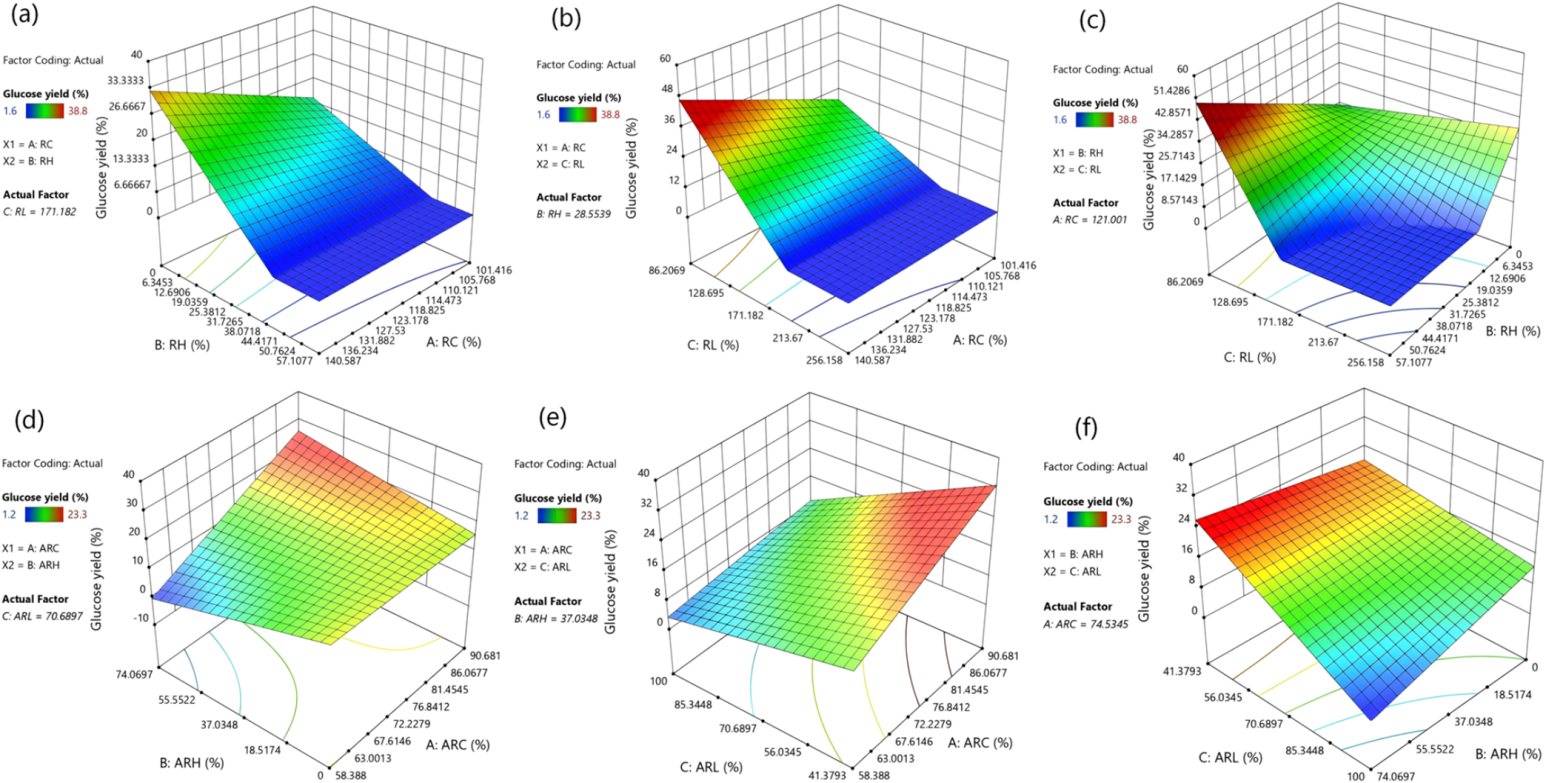
Response surface analysis of glucose yield as a function of composition retention metrics: (a–c) *R*_C_, *R*_H_, and *R*_L_; (d–f) AR_C_, AR_H_, and AR_L_.

Figure 3(a) indicates that the high-yield region is anchored at very low *R*_H_. The maximum observed yields occur at *R*_H_ = 2.14 (autohydrolysis, 38.8%) and *R*_H_ = 0 (acid, 37.6%), whereas yields can fall to 1.6% when *R*_H_ is high, such as 57.11 under autohydrolysis. This pattern is consistent with hemicellulose depletion serving as an accessibility switch that opens diffusion pathways and increases enzyme contact with cellulose.^16,41^ The surface also suggests diminishing returns once *R*_H_ becomes extremely low. Within the low-*R*_H_ band (approximately 0–3.6, spanning acid 0–1.47 and autohydrolysis 2.14–3.57), yields still vary widely, from 10.9–37.6% in acid and 25.2–38.8% in autohydrolysis, indicating that further reductions in *R*_H_ do not produce proportional gains. This behavior aligns with a shift in the dominant limitations from hemicellulose removal toward lignin-associated effects and residual structural constraints.^42,43^ In contrast, *R*_C_ contributes mainly as a conditional factor. Although *R*_C_ spans roughly 101.4–140.6 across the data set, similar *R*_C_ values correspond to markedly different yields, for example *R*_C_ = 125.92 with 25.2% yield in autohydrolysis versus *R*_C_ = 125.26 with 37.6% yield in acid, indicating that cellulose enrichment alone does not determine hydrolysis performance.^44,45^

Figure 3(b) surface does not support a universal monotonic penalty with increasing *R*_L_. High yields occur at both low and very high *R*_L_ values, including 37.6% at *R*_L_ = 86.21 in alkaline pretreatment, 37.6% at *R*_L_ = 256.16 in acid pretreatment, and 38.8% at *R*_L_ = 223.15 in autohydrolysis. This reflects that, in acid and autohydrolysis, elevated *R*_L_ often arises from relative lignin enrichment after preferential carbohydrate solubilization, rather than from the absolute amount of lignin remaining as a barrier.^46^ Considerable yield spread persists within the high-*R*_L_ domains typical of acid and autohydrolysis (acid 200.49–256.16; autohydrolysis 161.08–227.09), where yields range from 2.0–37.6% and 1.6–38.8%, respectively. This indicates that *R*_L_ acts mainly as a modulator whose effect depends on the concurrent carbohydrate state, rather than a standalone discriminator.^28,44^ At comparable mid–high *R*_C_ levels (approximately 120–130), the best yields are reached under very different lignin states across routes, reinforcing that the mapping between “lignin fraction” and hydrolysis outcome is route-conditioned by how pretreatment alters lignin distribution and surface chemistry.^47,48^

Figure 3(c) shows a much stronger gradient along *R*_H_ than along *R*_L_ over most of the composition space. Moving from high *R*_H_ (up to 57.11 in autohydrolysis) to very low *R*_H_ (down to 0 in acid and 2.14 in autohydrolysis) coincides with a shift from yields as low as 1.6–2.0% to maxima of 37.6–38.8%, even while *R*_L_ remains high in the acid/autohydrolysis domain (161–256). This indicates that hemicellulose depletion primarily governs accessibility and therefore sets the baseline for high hydrolysis.^49,50^ Substantial yield variation remains even at similarly low *R*_H_, for example acid reaches 37.6% at *R*_H_ = 0 and *R*_L_ = 256.16 but shows lower yields such as 19.2% at *R*_H_ = 1.47 and *R*_L_ = 244.33, indicating that lignin-related factors and structural rearrangements separate high from moderate performance once accessibility is broadly improved.^51,52^ Alkaline pretreatment occupies a distinct low-*R*_L_ region (86.21–161.08) with moderate *R*_H_ (16.22–36.39) yet still reaches the overall high end (37.6% at *R*_H_ = 16.22 and *R*_L_ = 86.21), consistent with effective lignin removal compensating for less extreme hemicellulose depletion.^53,54^

Figure 3(d) makes the digestibility–retention trade-off explicit by expressing performance on a raw-feedstock basis rather than on recovered-solid composition alone. Although dilute-acid and autohydrolysis can reach similarly high pretreated-basis glucose yields (up to 37.6% and 38.8%, respectively), their raw-basis yields are constrained to 1.30–19.70% (acid) and 1.20–23.30% (autohydrolysis), consistent with the associated solid-yield ranges of 48.2–66.3% and 59.8–77.1%. This indicates that high enzymatic digestibility can be offset by carbohydrate losses and mass removal, and AR_C_/AR_H_ capture this penalty directly.^55,56^ In addition, once hemicellulose retention approaches its lower bound (acid reaches AR_H_ = 0; autohydrolysis reaches AR_H_ = 3.57), raw-basis yield still varies widely (acid up to 19.70%), indicating that feedstock-level performance is then governed more by how much carbohydrate remains available on a raw basis than by further reductions in hemicellulose retention.^55,57^

Figure 3(e) shows that lignin retention becomes a meaningful, graded separator only in alkaline pretreatment. In the alkaline data set, AR_L_ spans 41.38–103.45% alongside raw-basis glucose yields of 8.70–18.00%, and the maximum raw-basis yield (18.00%) coincides with the minimum lignin retention (AR_L_ = 41.38%). This behavior is consistent with genuine lignin removal improving hydrolysis by reducing lignin shielding and limiting nonproductive enzyme adsorption on a feedstock basis.^58,59^ By contrast, dilute-acid and autohydrolysis frequently exhibit AR_L_ > 100%, so AR_L_ offers little within-route resolution for those data sets, consistent with lignin being largely unreduced and the apparent increases being dominated by relative concentration effects and measurement uncertainty.^60,61^

Figure 3(f) differentiates the route-specific pathways to higher raw-basis yield. For acid/autohydrolysis, extremely low AR_H_ values (acid down to 0; autohydrolysis down to 3.57) coexist with high AR_L_ (often >100%), while raw-basis yields remain bounded (≤19.70% and ≤23.30%, respectively). This indicates that accessibility gains from hemicellulose depletion are counterbalanced by mass loss and limited lignin reduction, constraining feedstock-level conversion.^62^ In alkaline pretreatment, raw-basis yield reaches the upper end (18.00%) at moderate AR_H_ values (33.79–57.36%) when AR_L_ is low (down to 41.38%), indicating that effective lignin removal can deliver favorable feedstock-level outcomes without requiring extreme hemicellulose depletion.^63^

## 4 Conclusion

This study formalizes a paired retention-metric framework to compare pretreatment routes for kenaf core in terms of reducing biomass recalcitrance, improving carbohydrate accessibility for enzymatic hydrolysis, and maximizing recoverable fermentable sugars on a feedstock basis. Two complementary descriptors are used together: the relative composition ratios (*R*_C_, *R*_H_, *R*_L_) to describe enrichment or depletion in the recovered solid, and the absolute retention indices (AR_C_, AR_H_, AR_L_) to quantify true component retention by incorporating solid recovery. When interpreted alongside glucose yields reported on both recovered-solid and raw-feedstock bases, the analysis highlights a digestibility–recovery trade-off, indicating that yield values are only meaningful when the reporting basis and solid yield are stated together. Route-resolved trends further show that hemicellulose depletion provides the most consistent indicator of increased glucose release, whereas lignin enrichment is not transferable as a pooled single-variable discriminator because its interpretation is pretreatment-chemistry dependent. Overall, the paired use of relative composition ratios and absolute retention indices separates mass-loss-driven apparent enrichment from true retention and supports pretreatment comparison and operating-condition selection toward higher feedstock-level sugar recovery through improved accessibility, while explicitly accounting for losses reflected in solid recovery.

## Abbreviations

LOESS: locally estimated scatterplot smoothing
R_C_: cellulose relative composition ratio
*R*_H_: hemicellulose relative composition ratio
R_L_: lignin relative composition ratio
AR_C_: cellulose absolute retention index
A*R*_H_: hemicellulose absolute retention index
AR_L_: lignin absolute retention index
HPLC: high-performance liquid chromatography
FPU: filter paper unit
